# Beyond weight: examining the association of obesity with cardiometabolic related inpatient costs among Canadian adults using linked population based survey and hospital administrative data

**DOI:** 10.1186/s12913-020-06051-2

**Published:** 2021-01-11

**Authors:** Neeru Gupta, Zihao Sheng

**Affiliations:** 1 Department of Sociology, University of New Brunswick, P.O. Box 4400, E3B 5A3 Fredericton, New Brunswick, Canada; 2 Department of Economics, Dalhousie University, Halifax, Canada

**Keywords:** Obesity, Cardiometabolic health, Hospital costs, Demographic and health surveys, National hospital discharge surveys, Data linkage

## Abstract

**Background:**

The global population has transitioned to one where more adults are living with obesity than are underweight. Obesity is associated with the development of cardiometabolic diseases and widely attributed to increased hospital resource use; however, empirical evidence is limited regarding obesity prevention to support hospital cost containment. This study aims to test for obesity in predicting hospitalization costs for cardiometabolic conditions among the Canadian population aged 45 and over.

**Methods:**

Data from the 2007−2011 Canadian Community Health Survey were linked to eight years of hospital discharge records. A cohort was identified of inpatients admitted for diabetes, hypertension, and other cardiometabolic diseases. Multiple linear regressions were used to investigate the association between obesity status and inpatient costs, controlling for sociodemographic and behavioural factors.

**Results:**

The target cohort included 23,295 admissions for cardiometabolic diseases. Although inflation-adjusted inpatient costs generally increased over time, compared with the non-obese group, living with obesity was not a significant predictor of differences in cardiometabolic-related resource use (0.972 [95% CI: 0.926–1.021]). Being female and rural residence were found to be protective factors.

**Conclusions:**

Obesity was not found in this study to be independently linked to higher cardiometabolic hospitalization costs, suggesting that actions to mitigate disease progression in the population may be more beneficial than simply promoting weight loss. Results amplified the need to consider gender and urbanization when formulating which levers are most amenable to adoption of healthy lifestyles to reduce impacts of obesogenic environments to the healthcare system.

## Background

The global population has transitioned to one where more people are living with obesity than are underweight [1]. In Canada, for example, obesity rates among adults nearly doubled over the past three decades, from 14% in 1978 to approximately 26% in 2013 [2]. These trends have been widely attributed to rapid urbanization and obesogenic environments, which may favour the adoption of unhealthy diets and sedentary behaviours [3, 4]. Obesity has been associated with higher incidence of chronic non-communicable diseases (NCDs) such as type 2 diabetes mellitus, ischemic heart disease, and related cardiometabolic complications and, in turn, increased risk of hospital admission, more frequent readmissions, and longer hospital stays [5–8]. At the same time, obesity is known to affect populations heterogeneously across clinical, sociodemographic, and behavioural characteristics [9–11]. Despite pervasive arguments that obesity is a key driver of growing healthcare expenditures for associated diseases, few studies have examined trends and determinants of hospital resource use and costs among persons living with obesity, especially ones including consideration of modifiable risk factors and drawing on nationally representative samples [8, 12–14]. Much of the literature on the hospital burden of NCDs focuses on the characteristics of healthcare systems, such as access and clinical caregiving, or on patients’ diagnosis-related complications, such as medical history and comorbidities [15–17]. It remains less well understood how sociodemographic and behavioural characteristics moderate the association between obesity and the hospital burden of cardiometabolic diseases.

Developments in the design of population-based studies have opened rich pathways for describing the role of sociodemographic factors on a range of health outcomes. In Canada, similar to many other countries, data linkages across population surveys and administrative information systems offer expanded opportunities to address research gaps on the sociodemographic correlates of common health problems and related impacts that would not be available from a single unlinked source [18–21]. The objective of this study was to examine the association of obesity with acute-care inpatient costs for cardiometabolic diseases, considering a range of other sociodemographic and behavioural characteristics. Acute inpatient care is one of the costliest categories to health systems. We take advantage of Canadian national household surveys linked with hospital administrative records to create a population-representative study cohort. Since obesity is rarely recorded as the primary reason for hospitalization, we hypothesized that trends in hospital costs for cardiometabolic diseases will reflect a net combination of obesity and other patient characteristics.

## Methods

### Data source

This study drew on a unique resource of multiple years of data from the Canadian Community Health Survey (CCHS) linked to the hospital Discharge Abstract Database (DAD). Conducted annually by Statistics Canada, the CCHS collects demographic and health data from a nationally representative sample of approximately 65,000 household respondents aged 12 and over. The DAD contains routinely collected demographic, administrative, and diagnostic data by fiscal year of hospital separation for all acute-care admissions in 12 of the country’s 13 jurisdictions (excluding the province of Quebec). Given the Canadian context of single-payer universal healthcare coverage, these data are considered a complete recording of all hospital stays. We pooled five years of CCHS cycles (2007−2011) with eight years of DAD datasets (2005/06−2012/13) to obtain sufficient sample sizes of cardiometabolic admissions. The survey and hospital data were matched using a probabilistic microdata linkage process based on individuals’ birthdate, sex, and residential postal code [21, 22]. We limited our analysis to the population aged 45 and over, when the risk of hospitalization for NCDs increases and healthcare costs may reflect greater cumulative exposure to obesity [13]. The deidentified linkable datasets were accessed in the Statistics Canada Research Data Centre located at the University New Brunswick, in accordance with data privacy and security protocols.

### Outcome measure: cost of acute‐care hospital stays

The outcome variable for this study was the cost estimate for each acute-care hospitalization for cardiometabolic-related conditions, based on DAD data. Admissions for cardiometabolic conditions are generally considered ambulatory care sensitive, that is, potentially avoidable by interventions in primary and community care [23–25]. The diagnostic data in the DAD are coded to the *International Classification of Diseases, 10th revision* (ICD-10-CA) [26]. Following research elsewhere [27], all hospitalizations were included where the primary diagnosis for the length of stay was for diabetes and other metabolic diseases (ICD-10-CA codes E10-E16, E70-E90), hypertensive diseases (I10I15), and cardio- and cerebro-vascular diseases (G08, G45, H34.0, H34.1, I20-I99). Using the methodology of the Canadian Institute for Health Information, hospital costs were estimated from aggregate information on the national cost of a standard stay applied to the resource intensity weight (RIW) for each DAD record [28]. The RIW reflects variations in expected resource use according to clinical and financial data, adjusted for patients’ age group and comorbidities, relative to total acute inpatient expenditures. We applied the latest 2012/13 standard cost (in Canadian dollars) to all data to control for inflation and other differences in relative cost-efficiency across time and locations. Since patients may have had more than one hospital stay over the period of observation, the total costs reflected both single admissions and readmissions (although readmissions were statistically rare).

### Predictor variables

The main predictor of interest was obesity status, based on self-reported height and weight information captured in the CCHS. We defined obesity status dichotomously from World Health Organization cut-offs for increased health risks: adults with a body mass index of at least 30 kg/m^2^ were classified as living with obesity. Women who were pregnant at the time of the survey were excluded. While self-reported height and weight data may be subject to measurement error, studies have supported the validity of their use, citing lack of evidence of statistical superiority of direct anthropometric measures in population-based assessments of health outcomes [29, 30]. It is noted that underweight has also been associated with higher hospital resource use and costs; however, this has been attributable in the literature to non-cardiovascular causes [31]. Moreover, persons with obesity represent a disproportionate number in the Canadian population and therefore have a much greater impact on healthcare resource utilization. Studies have further reported that nonobese overweight may have negative impacts on health and healthcare expenditures, but the associations tend to be more modest compared with the effects of obesity [32]. The clinical and public health interest lies in the magnitude and wide range of the impacts of obesity at the population level [11].

Previous research using (unlinked) CCHS data highlights a complex relationship of obesity with sociodemographic and lifestyle factors [33, 34]. We further considered several potentially confounding factors including individuals’ sex, age, place of residence (urban or rural), education (whether at least some postsecondary schooling was attained), physical activity (whether at least moderately active), dietary habits (number of daily consumptions of fruits and vegetables), tobacco use (whether currently smokes), and chronic disease status (whether reports having been told by a healthcare professional they had diagnosed diabetes, hypertension, or heart disease). Notably, some research has suggested that the association between obesity and hospital costs may differ between women and men [35, 36]; however, a surprising number of published studies do not consider sex in the study design.

### Analytical approaches

Our study conforms to the REporting of studies Conducted using Observational Routinely-collected health Data (RECORD) protocol [37]. Following a brief descriptive analysis of the study population drawing on findings from the CCHS, we present a flow chart illustrating the selection of the target cohort from linking the survey data with the routine hospital records. Counts were rounded and adjusted to respect data confidentiality controls. The final cohort for analysis was limited to respondents with non-missing survey data for height/weight and other predictors of interest. Although response rates for the CCHS are generally high, the nature of the distribution of non-responses for height/weight information is more uncertain [30].

Simple and multiple linear regressions were used to explore the role of obesity in explaining hospital costs for cardiometabolic diseases. The bivariate analyses considered key factors used to determine relative costs for acute inpatient care, notably, the length of stay (i.e., the number of days from admission to discharge) and the patients’ comorbidity level (i.e., an index of the cumulative hospital care requirements and resource impact of comorbidities, including both pre-existing and hospital-induced conditions) [38, 39]. The multiple regressions considered a range of patient-level sociodemographic and behavioural factors as potential confounders, and were further adjusted for the year of discharge. Individual characteristics at the time of the survey were assumed to represent those at the time of the hospital episode. All models were split by sex and used the logarithm of inpatient costs to reduce the effects of skewed data. The log transformation is widely applied in analyses of individual healthcare resource use and cost data; while certainly not the only methodological option, it was deemed appropriate for the present research given the policy interest, the absence of zero values (since only admitted patients were included), and the near-normality of the graphical representation following transformation (not shown due to privacy protocols) [40]. Regression parameters were estimated applying bootstrapped survey weights, to account for the complex CCHS sampling methods and ensure population representation of the results [41]. To ease interpretation, results are expressed in percentage terms of the predicted mean hospital cost difference by obesity status. All analyses were conducted using Stata v15, with confidence intervals (CIs) set at 95%.

## Results

### Study population

**Fig. 1:**
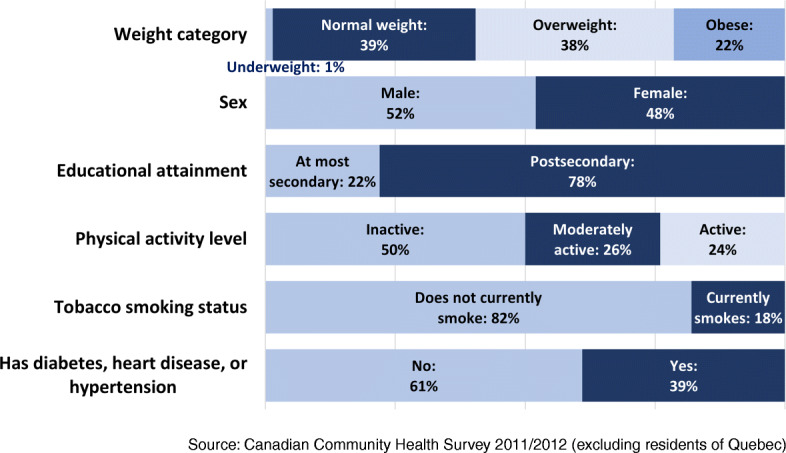
Percentage distribution of the Canadian population aged 45 and over by selected sociodemographic and behavioural characteristics

Among the Canadian population 45 and over, 22% (females: 23%; males: 20%) were living with obesity according to CCHS data (Fig. 1). Half (50%) were physically inactive and 39% reported having been diagnosed by a healthcare professional with diabetes (11%), hypertension (32%), or heart disease (9%). The frequency of fruits and vegetables consumption averaged 4.6 times a day (females: 5.0 times; males: 4.2 times), appreciably lower than national food intake recommendations for a healthy diet (not shown).

### Target cohort selection

**Fig. 2:**
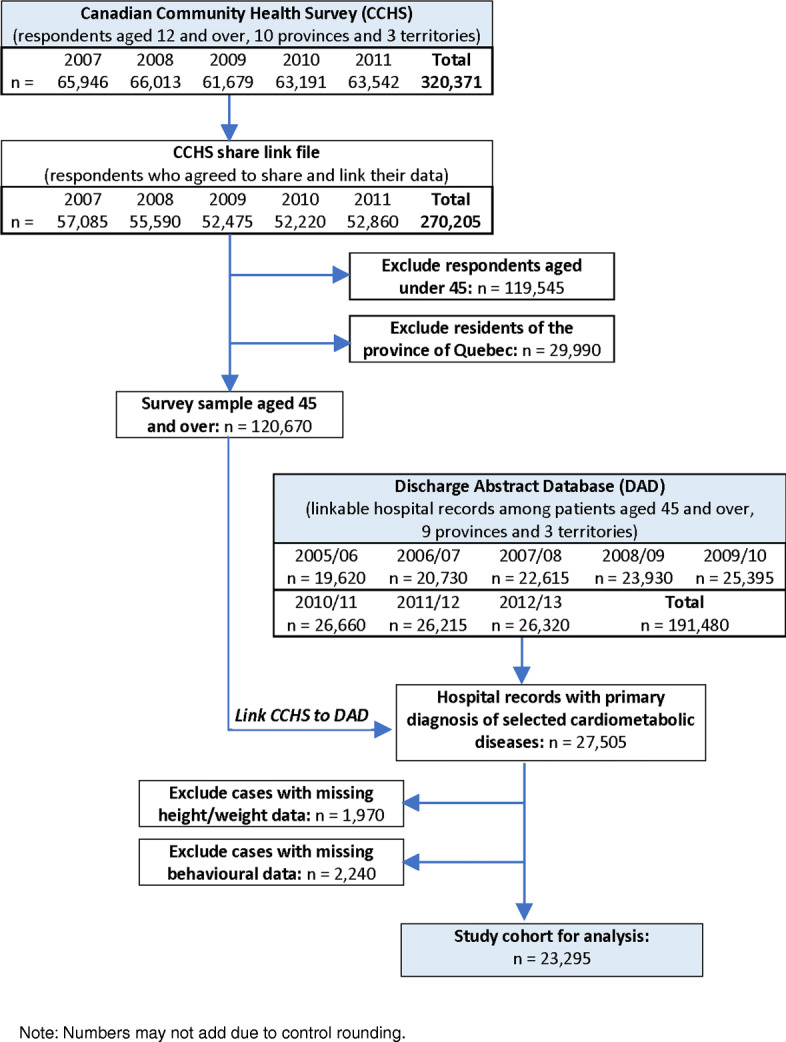
Flow chart for the creation of the study cohort from linked survey and hospital data

Five years of pooled CCHS data provided a sample of 320,371 household respondents, of whom 270,205 (84.3%) agreed to have their data shared and linked with other databases (Fig. 2). Among these respondents, 120,670 (44.7%) were aged 45 and over and eligible for data linkage to the DAD. Their data were linked to 27,505 hospital admissions for cardiometabolic diseases over the period of observation. Following data linkage, 25,535 inpatient records (92.8%) had valid information on obesity status and 23,295 (84.7%) had valid information on all survey-based characteristics of interest. Of the final study cohort, 10,800 (46.4%) were female and 12,485 (53.6%) were male.

### Obesity and hospital costs

Among the cohort under observation, based on simple regression analysis, obesity was not found to have influenced hospitalization costs for cardiometabolic diseases compared with the non-obese group: for both sexes combined, the relative mean cost difference was close to unity (0.970 [95% CI: 0.928–1.015]) (Table 1).

**Table 1:** Characteristics of acute-care hospitalizations for cardiometabolic diseases by obesity status of inpatients aged 45 and over, according to sex

	(1) Both sexes (*n*=25,535)			(2) Female (*n*=11,900)			(3) Male (*n*=13,635)		
	*β*	95% CI	*p*-value	*β*	95% CI	*p*-value	*β*	95% CI	*p*-value
**Mean length of stay (days)**									
Living with obesity	**6.5**	6.1–7.0	0.25	**7.1**	6.3–7.9	0.41	**6.1**	5.6–6.7	0.38
Not obese	**6.3**	6.0–6.5		**6.7**	6.3–7.2		**5.9**	5.6–6.2	
**Mean resource impact of comorbidities (comorbidity index)**									
Living with obesity	**9.1**	7.7–10.5	0.83	**9.7**	7.8–11.6	0.63	**8.7**	6.7–10.7	0.54
Not obese	**8.9**	8.1–9.8		**10.2**	8.9–11.5		**8.1**	6.9–9.2	
**Predicted mean cost difference (%)**									
Living with obesity	**97.0**	92.8–101.5	0.19	**101.8**	94.4–109.8	0.64	**93.7** *	88.5–99.3	0.03
Not obese	**--**			**--**			**--**		

Note: *=significantly different from the non-obese group (*p*<0.05); *CI* confidence interval. Predicted differences in mean cost by obesity status are based on simple log-linear regressions for all acute-care hospital stays (in constant Canadian dollars). All parameters are bootstrap weighted for population representation averaged over the period of observation

Source: 2007–2011 Canadian Community Health Survey linked to 2005/06–2012/13 Discharge Abstract Database

Considering the sex-specific models, in the opposite direction from expected, male inpatients living with obesity averaged only 94% of the hospital costs of their non-obese counterparts (0.937 [95% CI: 0.885–0.993]). No significant differences by obesity status in either mean length of stay or resource impact of comorbidities were observed which could potentially explain this pattern. Among women, no significant differences by obesity status in relative mean hospital cost (1.018 [95% CI: 0.944–1.098]) or other clinical characteristics of hospitalizations were found.

### Results of the multiple regression models

Results from the multiple linear regression for the total population revealed that acute-care costs for cardiometabolic conditions generally increased over time: compared with 2005/06, the cost averaged some 13% higher in 2009/10 (1.132 [95% CI: 1.043–1.229]) and 22% higher in 2012/13 (1.225 [95% CI: 1.108–1.353]) (Table 2, Model 1). Obesity was not found to be a significant predictor of cardiometabolic-related hospitalization costs (0.972 [95% CI: 0.926–1.021]). Rural residence was associated with approximately 7% less resource use compared with urbanity (0.927 [95% CI: 0.887–0.969]). As expected from established epidemiological patterns, inpatient costs increased significantly with age (1.004 [95% CI: 1.001–1.006]), while being female was found to be a protective factor (0.900 [95% CI: 0.860–0.942]). Patients’ lifestyle factors, including indicators of diet and tobacco use, did not influence hospital costs, after controlling for other sociodemographics.

**Table 2:** Adjusted coefficients (and 95% confidence intervals) from the multiple linear regressions for predictors of acute-care costs for cardiometabolic diseases among inpatients aged 45 and over, according to sex

	(1) Both sexes (*n*=23,295)			(2) Female (*n*=10,800)			(3) Male (*n*=12,495)		
	e^*β*^	95% CI	*p*-value	e^*β*^	95% CI	*p*-value	e^*β*^	95% CI	*p*-value
**Obesity status**									
Living with obesity (ref: Not obese)	**0.972**	0.926–1.021	0.25	**1.030**	0.949–1.117	0.48	0.934*	0.876–0.996	0.04
**Sex**									
Female (ref: Male)	0.900*	0.860–0.942	0.00	**--**			**--**		
**Age**									
Years	1.004*	1.001–1.006	0.00	1.004*	1.001–1.007	0.01	1.003*	1.000–1.007	0.03
**Place of residence**									
Rural (ref: Urban)	0.927*	0.887–0.969	0.00	0.918*	0.855–0.985	0.02	0.937*	0.889–0.989	0.02
**Educational attainment**									
Postsecondary (ref: At most secondary)	**0.983**	0.940–1.029	0.47	**0.965**	0.903–1.032	0.30	**0.998**	0.936–1.062	0.95
**Fruits and vegetables consumption**									
Times consumed daily	**1.007**	0.998–1.017	0.11	**1.002**	0.989–1.015	0.73	**1.011**	0.998–1.023	0.10
**Physical activity level**									
Moderately active to active (ref: Not active)	**0.966**	0.923–1.011	0.14	**0.961**	0.899–1.027	0.24	**0.968**	0.910–1.029	0.30
**Currently smokes tobacco**									
Yes (ref: No)	**1.042**	0.972–1.116	0.25	**1.055**	0.970–1.148	0.21	**1.031**	0.933–1.139	0.54
**Has diabetes, heart disease, or hypertension**									
Yes (ref: No)	1.079*	1.019–1.143	0.01	**1.064**	0.987–1.148	0.11	1.086*	1.003–1.176	0.04
**Year of hospital discharge**									
2006/07 (ref: 2005/06)	**1.017**	0.939–1.102	0.68	**1.036**	0.926–1.161	0.53	**1.008**	0.906–1.112	0.88
2007/08	**1.019**	0.933–1.112	0.68	**1.066**	0.923–1.232	0.38	**0.988**	0.888–1.098	0.82
2008/09	**1.087**	0.999–1.183	0.05	**1.117**	0.990–1.262	0.07	**1.073**	0.960–1.119	0.21
2009/10	1.132*	1.043–1.229	0.00	1.209*	1.065–1.372	0.00	**1.086**	0.978–1.206	0.12
2010/11	1.109*	1.022–1.204	0.01	1.114*	1.012–1.292	0.03	**1.092**	0.979–1.217	0.11
2011/12	1.156*	1.070–1.250	0.00	1.200*	1.059–1.361	0.00	1.132*	1.022–1.254	0.02
2012/13	1.225*	1.108–1.353	0.00	1.214*	1.085–1.357	0.00	1.243*	1.071–1.443	0.00

Note: *=*p*<0.05; *ref* reference group, *CI* confidence interval. Parameters are estimated from log-linear regressions of the cost of acute-care hospital stays (in constant Canadian dollars), and bootstrap weighted for population representation

Source: 2007–2011 Canadian Community Health Survey linked to 2005/06–2012/13 Discharge Abstract Database

Splitting the analysis by gender, living with obesity was associated with significantly lower cardiometabolic inpatient costs among men (0.934 [95%CI = 0.876–0.996]) (Table 2, Model 3). No significant association between obesity status and hospitalization costs was found among women (1.030 [95%CI = 0.949–1.117]) (Table 2, Model 2). Younger age and rural residence were associated with lower inpatient costs for each sex.

## Discussion

This study drew on multiple years of data from household surveys linked to acute-care hospital records to present, to our knowledge, the first Canadian population-representative investigation into the association of obesity status with cardiometabolic-related inpatient costs, as a summary indicator of disproportionate healthcare services resource use. We found that, while the inflation-adjusted costs for potentially avoidable hospitalizations for cardiometabolic diseases have been increasing over time, living with obesity was not distinguished as a significant cost predictor compared with those who were not obese (0.972 [95% CI: 0.926–1.021]) among inpatients aged 45 and over, after controlling for a range of sociodemographic, behavioural, and health status indicators.

This result is consistent with research elsewhere indicating that the population living with obesity is not homogenous [10], and that obesity may not be significantly associated with higher healthcare costs independently of other behavioural factors associated with future risk of diabetes, hypertension, and related complications [42]. Obesity is widely defined in the literature in relation to body mass index; however, there is a lack of universally-accepted definition and metric for metabolically healthy obesity as an important subgroup, which different studies have assessed as representing from 6–75% of the population living with obesity [43, 44]. The present findings thus support arguments that population-based actions to reduce the burden of obesity may be better placed focusing on interventions to reduce the risk of disease progression, rather than simply on reducing excess body weight at a given point in time [42, 45].

As expected, survey respondents reporting having been diagnosed with diabetes, heart disease, or hypertension averaged higher cardiometabolic-related hospital costs; it is possible, however, that some patients may have received their diagnosis during a hospital stay after interview. Urban residence was found to be associated with higher hospital costs compared with rural residence. Evidence on urban–rural differences in the hospital burden of obesity-associated NCDs is generally inconclusive; rural residency was found to have a negative association with the risk of diabetes-attributable readmissions in a study from the United States [46] but no significant association according to findings from Canada [19]. Somewhat unexpectedly, we did not find a significant association between smoking status and hospital costs. This result may be explained in part by survival selection bias inherent to observational studies among aging populations, due to higher pre-hospitalization mortality associated with tobacco use or other uncontrolled cardiometabolic risk factors [47, 48].

Based on split-sex regressions, we further found that obesity was independently associated with lower cardiometabolic hospital costs among men but not among women. Such results amplify the need to consider sex and gender when formulating which policy and practice levers are most amenable to the adoption of healthy lifestyles, notably in terms of weight stigma as a potential additional contributor to health disparities, especially among women. Obesity is identified in many societies as a highly stigmatizing personal attribute, one that may adversely affect medical compliance and implementation of healthy behaviours, and the risk of perceived interpersonal discrimination has been found to vary by gender and socioeconomic status [49].

Some limitations to this study should be noted. First, we focused on acute-care hospital costs, among the most expensive to health systems. While inpatient costs are considered essentially complete in this context of publicly-funded universal coverage, our analysis did not account for other direct and indirect costs for the treatment and management of NCDs, including services in primary and community-based care settings, emergency department visits that did not lead to admission, patients’ out-of-pocket expenses such as non-insured medical equipment, or sickness absence from work, among others [50]. Second, obesity was categorized dichotomously from a cut-point for body mass index, rather than on a scale of body composition. The obesity phenotype is changing over time in Canada, with measures of body fat and fat distribution potentially contributing more to cardiometabolic morbidity in recent years than obesity prevalence [51]. Moreover, the need for more research on healthcare resource use among underweight patients has been identified [31]. Third, we only considered hospital costs for selected cardiometabolic diseases. Various other health problems that may result in hospitalization have been associated with obesity, such as osteoarthritis, back problems, and depression [5]. Fourth, obesity status was captured only once, at interview, which may raise concerns of reverse causality [18]. On the other hand, given the wider socioenvironmental influences hindering weight loss and its maintenance among adults, significant changes in weight categories in the shorter-term would seem unlikely.

## Conclusions

Much of the health services literature on understanding the drivers of hospital costs has focused on measures of hospital infrastructure and healthcare system characteristics [15]. Advances in integrating different types of data platforms are supporting enhanced understanding of the trends, differentials, and risk factors underlying health outcomes and morbidity processes, especially among aging populations. This study adds to the literature recognizing the value of using linked population-based survey and administrative datasets to support research on obesity as a hypothesized key risk factor for hospital resource use and costs, since weight and other patient characteristics are not routinely captured in administrative hospital discharge records. Obesity was not found to be a significant driver of cardiometabolic-related hospital costs among the Canadian population aged 45 and over, after controlling for sociodemographic and behavioural factors, thus raising questions as to whether obesity prevention alone is an efficient hospital cost containment measure. The present findings fuel increasingly widespread arguments to prioritize interventions aimed at mitigating the risk of chronic disease progression, rather than simply focusing on weight loss to reduce the burden of obesity.

## Data Availability

The data that support the findings of this study are available from Statistics Canada but restrictions apply to the availability of these data, which were used under license for the current study, and so are not publicly available.

## Abbreviations

CCHS: Canadian Community Health Survey
CI: confidence interval
DAD: Discharge Abstract Database
ICD-10: International Classification of Diseases, 10th revision
NCD: non-communicable disease
RECORD: REporting of studies Conducted using Observational Routinely-collected health Data
RIW: resource intensity weight
